# Expression of DLL3 and SEZ6 in the Spectrum of Neuroendocrine Neoplasia

**DOI:** 10.1007/s12022-025-09873-0

**Published:** 2025-07-23

**Authors:** Iqbal Ahmed, Amr Mohamed, Omid Savari, Yue Xue, Sylvia L. Asa

**Affiliations:** 1 Department of Pathology, University Hospitals Cleveland, Case Western Reserve University, Institute of Pathology, 11100 Euclid Avenue, Room 204, Cleveland, OH USA; 2 Seidman Cancer Center, University Hospitals Cleveland, Case Western Reserve University, CLEVELAND, USA

**Keywords:** DLL3, SEZ6, Neuroendocrine neoplasms, Neuroendocrine tumor, Neuroendocrine carcinoma, Paraganglioma

## Abstract

Delta-like protein 3 (DLL3), a Notch ligand, has been identified in high-grade small- and large-cell lung carcinomas and prostate neuroendocrine carcinomas (NECs). SEZ6 (Seizure-related 6 homolog), a membrane-associated protein, has also been identified neuroendocrine neoplasms (NENs). Both DLL3 and SEZ6 are targets of novel antibody–drug conjugates (ADCs). Their expression in the broader family of NENs remains to be clarified. We examined a series of NENs of all types as well as several non-neuroendocrine neoplasms using immunohistochemistry for DLL3 and SEZ6. Staining was scored with the semi-quantitative assessment of intensity and percentage of stained cells that yields a histoscore (H-score from 0 to 300). We identified strong expression of DLL3 in all lung NECs (average H-score 180) and SEZ6 in 1 of 3 (H-score 70). Merkel cell carcinomas (*n* = 13) expressed DLL3 strongly and diffusely (H-score 178, range 10–300) and 10 of 13 had positivity for SEZ6 (H-score 128). Both DLL3 and SEZ6 were expressed in medullary thyroid carcinomas (10 of 11 cases, H-scores 199 for DLL3 and 224 for SEZ6). Two thymic neuroendocrine tumors (NETs) had weak expression of DLL3 (H-score 20) and SEZ6 (H-score 70). Nine of 11 lung NETs expressed DLL3 (H-score 191), while only two had focal weak staining for SEZ6 (H-score 45). DLL3 was negative in nine of 11 pancreatic NETs; two grade 3 pancreatic NETs had variable weak positivity (H-score 5). In contrast, seven of 11 pancreatic NETs expressed SEZ6 (H-score 45). DLL3 was positive in two pancreatic NECs (H-score 197), and SEZ6 was positive in 1 (H-score 60). One of 13 gastric NETs, a metastatic grade 3 tumor, expressed both DLL3 and SEZ6 (H-score 200 for each) and one other expressed SEZ6 at lower levels. A gastric NEC was weakly positive for both markers (H-scores 50 and 40). All 10 duodenal, 10 ileal, and nine rectal NETs were negative for DLL3; eight duodenal, six ileal, and four rectal NETs expressed SEZ6 (average H-scores 206, 78 and 45, respectively). Among 10 appendiceal NETs, two expressed DLL3 focally and weakly (H-score 45); eight were positive for SEZ6 (H score 109). Duodenal NECS (*n* = 2) were negative for DLL3; one duodenal NEC expressed SEZ6 (H-score 110). Among five colonic NECs, two expressed DLL3 (H-score 50) and one expressed SEZ6 (H-score 40). Pituitary NETs also expressed DLL3 with eight of 18 positive (H-scores from 10 to 180), and 11 of 18 expressed SEZ6 (average H-score 65). Three of 20 paragangliomas expressed DLL3 weakly (H-score 43), and six expressed SEZ6 (H-score 73). One of four parathyroid carcinomas expressed DLL3 weakly (H-score 30), and all four were negative for SEZ6; five parathyroid adenomas were negative for both. In 43 non-neuroendocrine neoplasms of the GI tract, pancreas, and liver and 10 non-neuroendocrine thyroid carcinomas, there was only weak focal reactivity for DLL3 in three and two cases, respectively, and for SEZ6 in one case each. These results suggest that expression of DLL3 and SEZ6 varies among NENs of almost all types. Expression appears to be limited primarily to NENs and is rarely seen only focally and weakly in non-NENs. The presence of one or both of these antigens offers a novel approach to the treatment of patients with neuroendocrine neoplasms across the entire spectrum.

## Introduction

Neuroendocrine neoplasms (NENs) represent a diverse group of tumors arising from neuroendocrine cells throughout the body. These tumors exhibit varying degrees of differentiation and biological behavior, ranging from well-differentiated neuroendocrine tumors (NETs) to poorly differentiated neuroendocrine carcinomas (NECs). Recent advancements in targeted therapies have highlighted the importance of identifying novel biomarkers for these tumors. Two proteins of particular interest are Delta-like protein 3 (DLL3) and Seizure-related 6 homolog (SEZ6).

DLL3 is a Notch ligand that is characterized by a DSL domain, EGF repeats, and a transmembrane domain [1]. Expression of DLL3 is highest in fetal brain and paraxial mesoderm, and mutations in the *DLL3* gene cause the autosomal recessive genetic disorder Jarcho-Levin syndrome characterized by spondylocostal dysostosis [2, 3]. Expression of DLL3 is known to be a feature of pulmonary high-grade small-cell and large-cell neuroendocrine carcinomas and high-grade prostate neuroendocrine carcinomas as well as other neuroendocrine neoplasms [4–6].

SEZ-6 is specifically expressed in the adult brain and testis [7] where it encodes a transmembrane protein that has putative roles in cell–cell recognition and signaling involved in brain development, synapse formation, and neuronal function [8]. SEZ6 is a membrane-associated protein. It has been studied primarily in the context of neurobiology but is gaining interest in cancer research. It too has been identified in neuroendocrine neoplasms including small-cell lung carcinoma [9] and medullary thyroid carcinoma [10].

Both DLL3 and SEZ6 have emerged as targets for novel antibody–drug conjugates (ADCs), making them promising candidates for targeted therapy in NENs. Bispecific antibodies targeting DLL3 [11] have been used for pulmonary neuroendocrine neoplasms [9], and a similar bispecific antibody approach has been used to target SEZ6 [9, 12]. However, their expression patterns across the spectrum of neuroendocrine neoplasia remain to be fully elucidated.

We investigated whether these biomarkers are expressed in a large cohort of neuroendocrine tumors of all types based on location and grade, as well as in non-endocrine tumors.

## Materials and Method

### Cohort and Clinicopathologic Characteristics

A retrospective review of the institutional pathology files was performed to identify NENs from various anatomical sites and compared with non-neuroendocrine neoplasms. A total of 213 formalin-fixed, paraffin-embedded tissue samples were obtained from the pathology archives of University Hospitals Cleveland Medical Center. The study was conducted with institutional research ethics approval.

Tumors were classified using the fifth edition WHO guidelines [13–16].

### Immunohistochemistry Procedures and Assessment

Immunohistochemical staining for DLL3 and SEZ6 was performed on a Ventana Benchmark Ultra stainer using the Ultraview DAB protocol. DLL3 was localized using the prediluted Ventana SP347 antibody, and SEZ6 was examined using the Abcam EPR28518-55 recombinant IgG clone at 1:500 dilution. The positive control for DLL3 was a small-cell lung carcinoma, and the positive control for SEZ-6 was a pancreas.

The expression of DLL3 and of SEZ6 was categorized as negative (0) or positive with semi-quantitative scoring for intensity (1 = weak, 2 = moderate, 3 = strong) and percentage of stained cells (1–100%) using light microscopy. The product of these two parameters yields a histoscore (H-score) that ranges from 0 to 300. In tumors with variable expression, multiple areas were examined and the average was used for the tumor. All slides were reviewed by two authors (IA and SLA), and any cases with discrepancies were examined together to achieve consensus.

## Results

We identified 160 NENs and 53 non-NENs to examine the expression of DLL3 and SEZ6 using immunohistochemistry. The staining in positive cases varied from strongly and diffusely positive to focal strong or weak mixed cytoplasmic and membranous staining, and a few cases had diffuse weak positivity. The results of stains in the 160 NENs and 53 non-NENs are summarized in Table 1.

**Table 1:** Expression of DLL3 and SEZ6 in neuroendocrine neoplasms

Tumor type	DLL3		SEZ6	
	Number of cases	Average H-score with range in parentheses*	Number of cases	Average H-score with range in parentheses*
Pituitary NET	8/18	50 (10–180)	11/18	65 (10–200)
Thymic NET	2/2	20 (for both)	2/2	70 (60–80)
Medullary thyroid carcinoma	10/11	199 (50–300)	10/11	224 (120–300)
Parathyroid adenoma	0/5	-	0/5	-
Parathyroid carcinoma	1/4	30	0/4	-
Lung NET	9/11	191 (5–300)	2/11	45 (10–80)
Lung NEC	3/3	180 (120–270)	1/3	70
Gastric NET	1/13	200	2/13	160 (120–200)
Gastric NEC	1/1	50	1/1	40
Duodenal NET	0/10	-	8/10	206 (50–300)
Duodenal NEC	0/2	-	1/2	110
Ileal NET	0/10	-	6/10	78 (30–160)
Appendix NET	2/10	35 (30–40)	8/10	109 (70–260)
Colorectal NET	0/9	-	4/9	45 (10–100)
Colorectal NEC	2/5	50 (10–90)	1/5	40
Pancreatic NET	2/11	5 (for both)	7/11	45 (5–100)
Pancreatic NEC	2/2	197 (100–294)	1/2	60
PPGL	3/20	43 (10–100)	6/20	73 (5–160)
Merkel cell carcinoma	13/13	178 (10–300)	10/13	128 (30–200)
Non-neuroendocrine thyroid neoplasms	2/10	10 (for both)	1/10	10
Non-neuroendocrine gastrointestinal, liver and pancreatic neoplasms	3/43	30 (20–50)	1/43	10

^*^Calculated for positive cases

### Pituitary Neuroendocrine Tumors

**Fig. 1:**
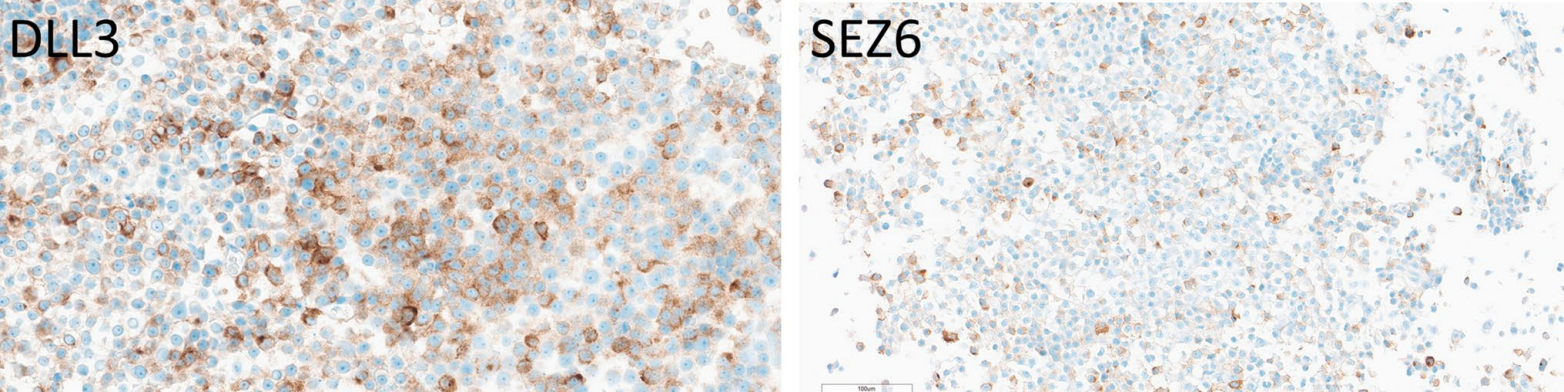
Immunohistochemical localization of DLL3 and SEZ6 in pituitary neuroendocrine tumors. A recurrent sparsely granulated silent corticotroph tumor stains strongly for DLL3 in about 60% of tumor cells (H-score 180) and has variable positivity for SEZ6 (H-score illustrated 120)

Among the 18 pituitary neuroendocrine tumors examined, DLL3 expression was observed in eight tumors (44.44%) with average H-score 50, range 10–180, and SEZ6 expression was noted in 11 cases (61%), with average H-score 65, range 10–200. We examined tumors of the various cell lineages and subtypes (Table 2). Among six corticotroph tumors that included two densely granulated, three sparsely granulated, and one Crooke cell tumor, two were positive for DLL3 and three for SEZ6. The strongest expression of DLL3 was observed in a recurrent sparsely granulated silent corticotroph tumor that also had loss of ATRX (Fig. 1). The Crooke cell tumor was negative for both DLL3 and SEZ6. The PIT1-lineage tumors included two densely granulated and one sparsely granulated somatotroph tumors, two sparsely granulated lactotroph tumors, two mammosomatotroph tumors, and one immature PIT1-lineage tumor. Among these eight tumors, six expressed DLL3 focally and weakly and seven expressed SEZ6; interestingly, the aggressive immature PIT1-lineage tumor was negative for DLL3 but expressed SEX6 diffusely (H-score 200). Gonadotroph tumors (*n* = 4) were negative for DLL3 and only one expresses SEZ6 (H-score 160).

**Table 2:** DLL3 and SEZ6 immunoreactivity in pituitary neuroendocrine tumors

Pituitary tumor type	DLL3		SEZ6	
	Number of cases	Average H-score with range in parentheses*	Number of cases	Average H-score with range in parentheses*
Corticotroph tumor	2/6	105 (30–180)	3/6	70 (10–190)
Somatotroph tumor	2/3	30 (10–50)	2/3	15 (10–20)
Lactotroph tumor	2/2	15 (10–20)	2/2	10 (for both)
Mammosomatotroph tumor	2/2	50 (10–90)	2/2	50 (20–80)
Immature PIT1 lineage tumor	0/1	-	1/1	200
Gonadotroph tumor	0/4	-	1/4	160

^*^Calculated for positive cases

### Thymic Tumors

**Fig. 2:**
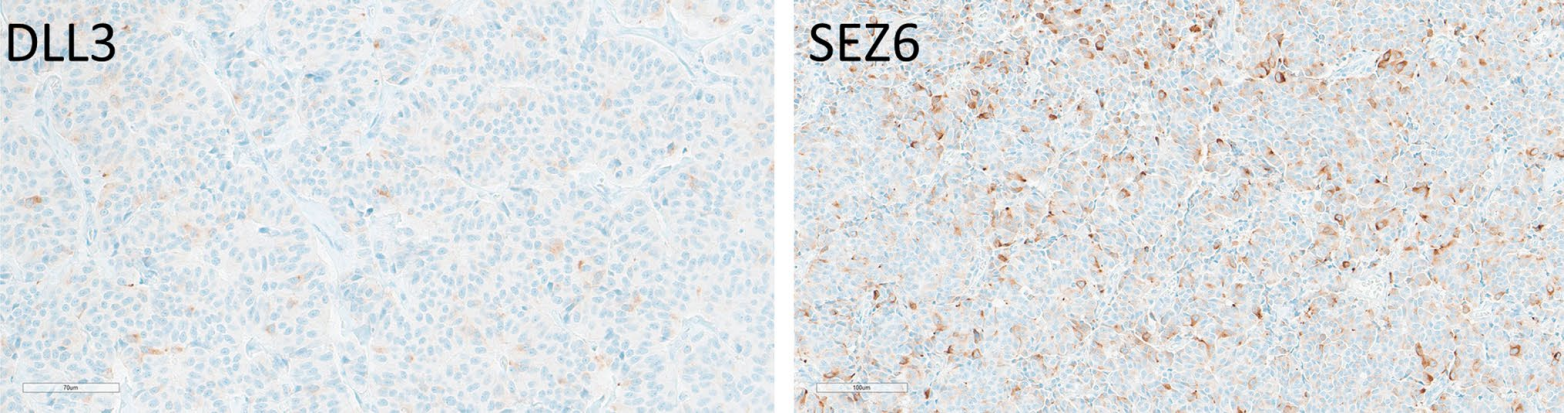
Immunohistochemical localization of DLL3 and SEZ6 in thymic neuroendocrine tumors. A grade 2 thymic NET has weak positivity for DLL3 (H-sore 20) and variable staining for SEZ6 (H-score 80)

Two well-differentiated grade 2 thymic neuroendocrine tumors had positive staining for both DLL3 and SEZ6 (Fig. 2), with moderate intensity in about 10% of cells for DLL3 (H-score 20 for both), and 20% strong and 40% moderate positivity of cells for SEZ6 (average H-score 70, range 60–80).

### Medullary Thyroid Carcinomas

**Fig. 3:**
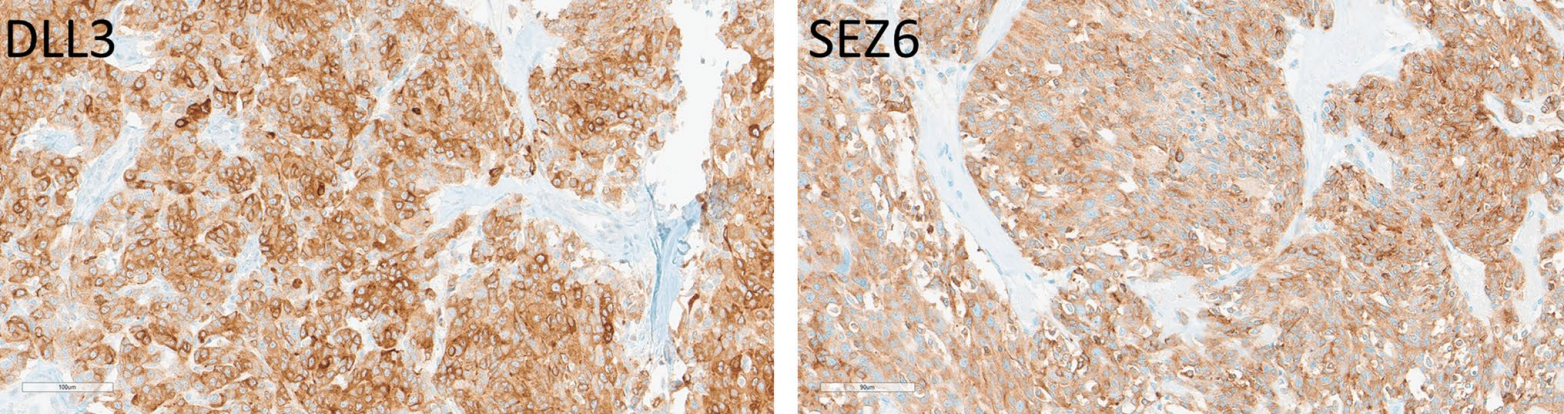
Immunohistochemical localization of DLL3 and SEZ6 in medullary thyroid carcinomas. A high-grade medullary thyroid carcinoma expresses DLL3 strongly and diffusely (H-score 300) and SEZ6 strongly and in most tumor cells (H-score 285)

Medullary thyroid carcinomas demonstrated strong expression of both markers. Ten of 11 cases (91%) were positive for both DLL3 and SEZ6 (Fig. 3), with staining intensities ranging from weak to strong in 20–100% of tumor cells for DLL3 (average H-score 199, range 50–300), and with moderate to strong staining intensities in 40–100% of cells for SEZ6 (average H-score 224, range 120–300). Staining was concordant in primary and metastatic foci. Seven tumors were low grade, and four were high grade; while the high-grade tumors tended to have high H-scores (DLL3 300, 300, 285, and 100; SEZ6 285, 200, 200, and 120), there was no correlation with staining intensity or H-score, since some of the low-grade tumors also had high H-scores; however, the only micromedullary thyroid carcinoma was negative for both DLL3 and SEZ6.

### Parathyroid Neoplasms

**Fig. 4:**
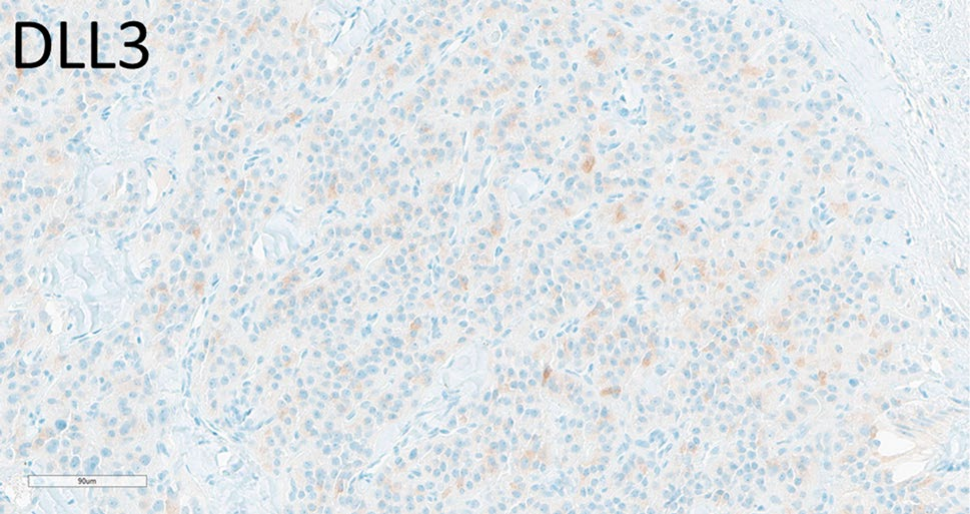
Immunohistochemical localization of DLL3 in parathyroid carcinoma. The only parathyroid tumor to exhibit positivity for DLL3 was this parathyroid carcinoma (H-score 30)

We examined five parathyroid adenomas, including a lipoadenoma, a chief cell adenoma, an oncocytic adenoma, and two clear cell adenomas; all these tumors were negative for both DLL3 and SEZ6. Among the four parathyroid carcinomas examined, one (25%) had positivity for DLL3 with weak staining in 30% of cells (H-score 30) (Fig. 4). All cases were negative for SEZ6.

### Lung Neuroendocrine Neoplasms

**Fig. 5:**
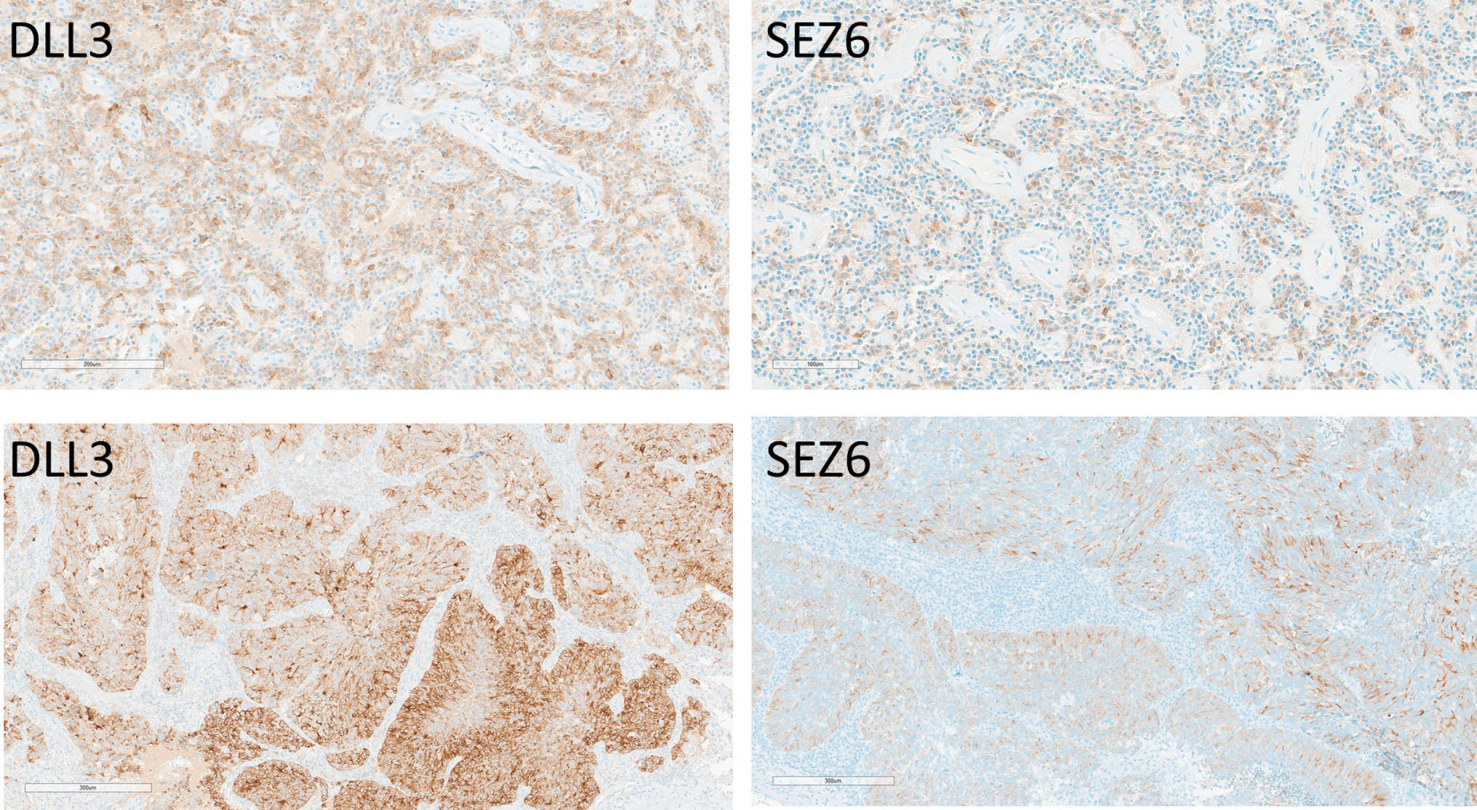
Immunohistochemical localization of DLL3 and SEZ6 in lung neuroendocrine neoplasms. A well differentiated lung NET (top) expresses DLL3 with variable intensity (H-score 190) and SEZ6 focally (H-score 80). A large-cell lung NEC (bottom) has somewhat heterogeneous intense staining for DLL3 (H-score 270) and variable staining for SEZ6 (H-score 70)

Well-differentiated lung NETs (*n* = 11) frequently expressed DLL3, with nine tumors (81.8%) showing positivity ranging from 5 to 100% of tumor cells (average H-score 191; range 5–300) (Fig. 5). SEZ6 expression was less common, observed in only two cases (18.2%) (average H-score 45, actual scores 10 and 80); DLL3 was strongly expressed in these two tumors with H-scores of 240 and 285) (Fig. 5).

Small-cell (*n* = 1) lung carcinoma expressed DLL3 moderately, and large-cell neuroendocrine carcinomas (*n* = 2) of the lung demonstrated strong DLL3 positivity (50–90% of cells; average H-score 180 combined, range 120 to 270) in all the tumors examined, including metastatic foci (Fig. 5). SEZ6 expression was seen in one large-cell NEC with weak positivity in 70% of cells (H-score 70) (Fig. 5) and not in small-cell lung carcinoma.

### Gastrointestinal Neuroendocrine Neoplasms

Gastric NETs (*n* = 13) were mostly negative for DLL3; only one metastatic well-differentiated NET had moderate staining in 100% of the cells (H-score 200). SEZ6 positivity was observed in two cases, one type 1 ECL cell NET showing weak to moderate staining in 80% of the cells (H-score 120), and one metastatic well-differentiated grade 3 NET showing moderate staining in 100% of the cells (H-score 200). One gastric NEC was positive for both markers (H-score for DLL3 50 and for SEZ6 40).

**Fig. 6:**
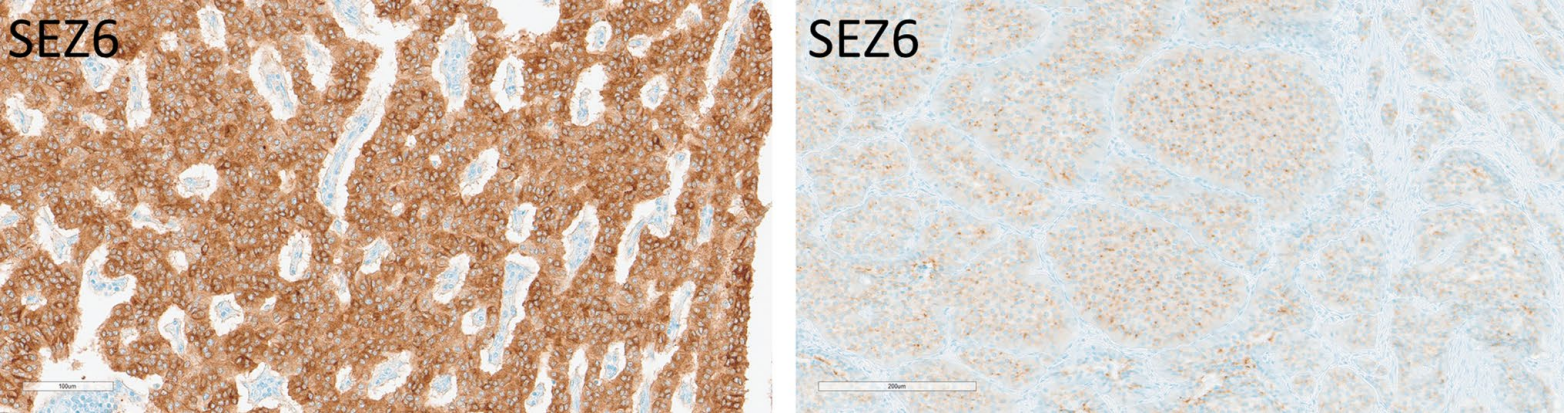
Immunohistochemical localization of SEZ6 in gastrointestinal neuroendocrine neoplasms. A duodenal gastrin-producing NET (left) has diffuse and strong positivity for SEZ6 (H-score 300). An appendiceal EC cell NET expresses SEZ6 weakly and diffusely (H-score 100 illustrated)

Duodenal (*n* = 10) and ileal EC cell NETs (*n* = 10) were negative for DLL3. Eight of 10 (80%) duodenal NETs were weakly to strongly positive for SEZ6 in 50–100% of cells (average H-score 206; range 50–300) (Fig. 6). SEZ6 expression in ileal NETs was overall weaker, with and average H-score of 78 (range 30–160) and did not correlate with tumor grade. The two duodenal NECs were negative for DLL3, and one large-cell NEC was weakly to moderately positive for SEZ6 in 80% of the cells (H-score 110) (not shown).

Appendiceal NETs (*n* = 10) included four L cell NETs and six EC cell NETs. Only two of 10 cases (one L cell NET and one EC cell NET) were weakly positive for DLL3 in 30 to 40% of the cells (average H-score 35, actual score 30 and 40). Eight of 10 cases were weakly to strongly positive for SEZ6 in 50–100% of the cases (average H-score 109; range 70–260) (Fig. 6). Neither stain was preferentially associated with tumor grade.

Colorectal NETs (*n* = 9) included six L cell and three EC cell tumors. One was metastatic to liver. All were DLL3-negative, with variable weak SEZ6 expression in four cases that included two L cell tumors (44.44%) (average H-score 45, range 10–100) (not shown). Of five colonic NECs, including adenocarcinoma with neuroendocrine differentiation, two were weakly to strongly positive for DLL3 (average H-score 50; actual scores 10 and 90). The only NEC positive for SEZ6 was a poorly differentiated adenocarcinoma with variable neuroendocrine differentiation; it was strongly positive for DLL3 and was also moderately positive for SEZ6 in 20% of the cells (H-score 40).

### Pancreatic Neuroendocrine Neoplasms

**Fig. 7:**
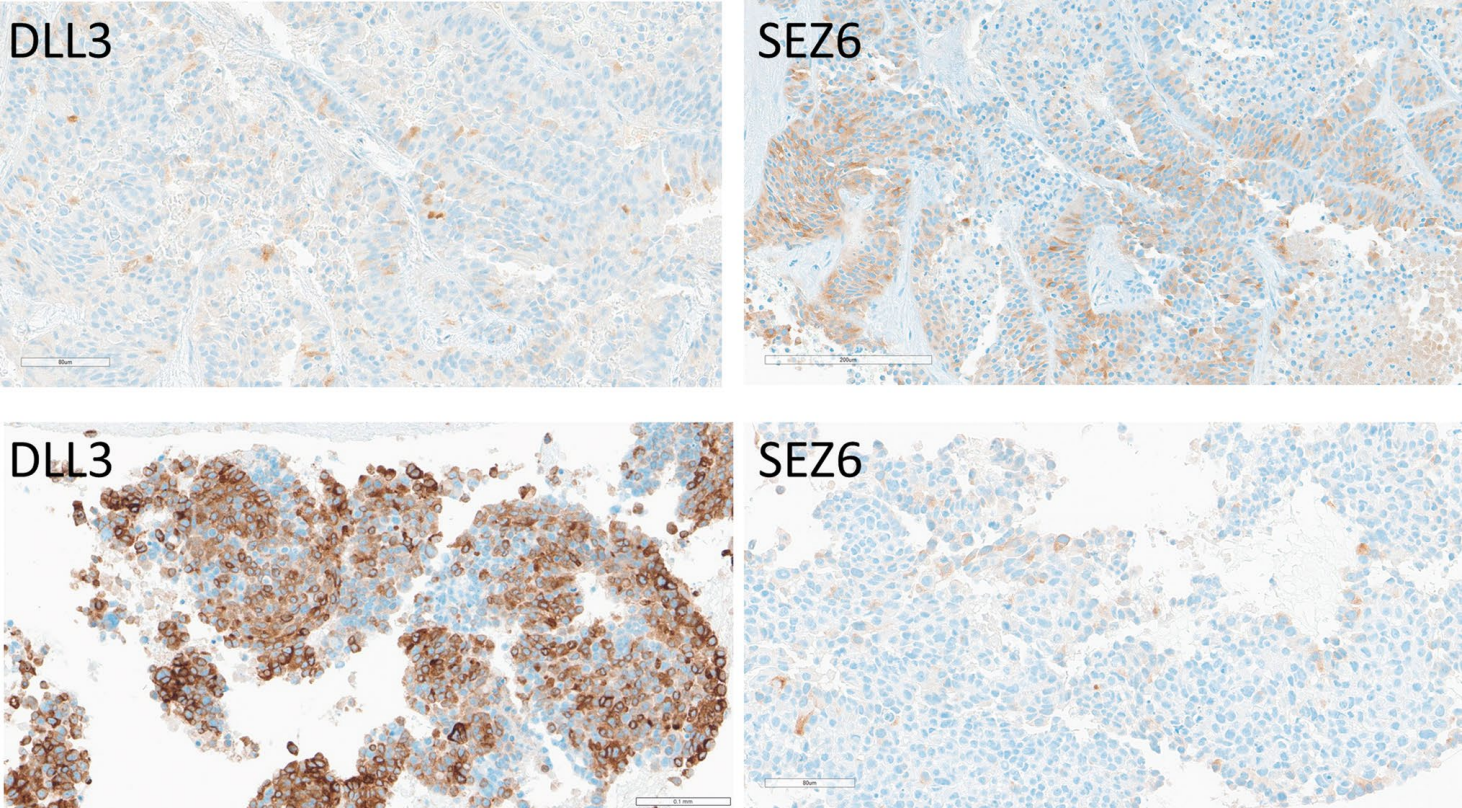
Immunohistochemical localization of DLL3 and SEZ6 in pancreatic neuroendocrine neoplasms. A grade 3 well-differentiated pancreatic NET (top) expresses DLL3 only very focally (H-score 5) and SEZ6 focally with some moderate and some strong intensity (H-score 100). A large-cell pancreatic NEC (bottom) has somewhat heterogeneous intense staining for DLL3 (H-score 270) and scant weak staining for SEZ6 (H-score 60)

**Fig. 8:**
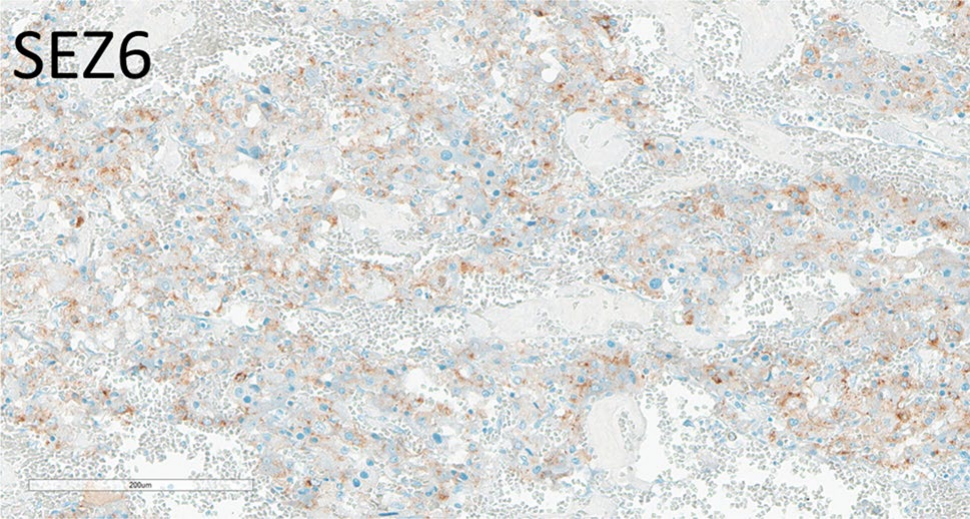
Immunohistochemical localization of SEZ6 in paragangliomas. An SDH-deficient extra-adrenal paraganglioma exhibits moderate positivity for SEZ6 in about 50% of tumor cells, yielding an H-score of 100

Well-differentiated pancreatic NETs (*n* = 11) were largely DLL3-negative, with two G3 NETs showing focal weak positivity in 5% of the tumor cells (H-score 50 for both tumors) (Fig. 7). SEZ6 expression was more common, observed in seven cases (54.5%) with varying intensities, including strong staining in G3 tumors (H-scores ranging from 5 to 100; average: 45) (Fig. 8). While G3 tumors were more frequently positive and had stronger positivity, there was no strict correlation between tumor grade and SEZ6 reactivity.

Pancreatic NECs (*n* = 2) showed strong DLL3 positivity (50–98% of cells) (average H-score 200) (Fig. 8), and one case had strong SEZ6 expression in 20% of the tumor cells (H-score 60) (Fig. 7).

### Paragangliomas and Pheochromocytomas

Paragangliomas (*n* = 20, of which seven were adrenal pheochromocytomas) included three SDH-deficient tumors, one case of multifocal adrenal pheochromocytoma, one associated with VHL syndrome, one composite pheochromocytoma and ganglioneuroma in a patient with neurofibromatosis, and two metastatic tumors. Three extra-adrenal paragangliomas had weak to moderate DLL3 positivity in 10–100% of the tumor cells (average H-score 43; range 10–100); one was metastatic, one was SDH-deficient, and the other was non-metastatic. SEZ6 expression was variable in six cases, with average H-score of 73 (Fig. 8), range (5–160). Only one SDH-deficient paraganglioma expressed SEZ6.

### Merkel Cell Carcinomas

**Fig. 9:**
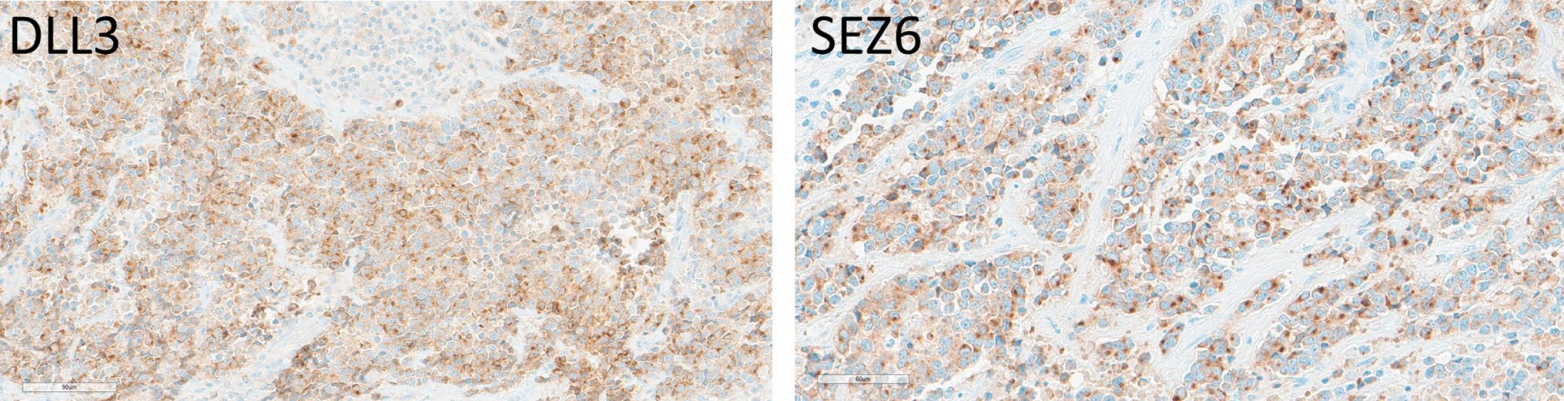
Immunohistochemical localization of DLL3 and SEZ6 in Merkel cell carcinomas. DLL3 expression is 60% moderate and 40% strong, providing an H-score of 240. SEZ6 expression is moderate in 90% of tumor cells yielding an H-score of 180

All 13 cases of Merkel cell carcinoma including primary and metastatic disease showed weak to strong DLL3 positivity in 10–100% of cells (average H-score 178, range 10–300) (Fig. 6). SEZ6 expression was variable, with 10 of 13 cases (77%) showing positivity in 30–90% of cells (average H-score 128, actual scores 30 and 200) (Fig. 9).

### Non-neuroendocrine Neoplasms

We examined 53 non-neuroendocrine neoplasms including moderately and poorly differentiated colonic (*n* = 10) and pancreatic (*n* = 11) adenocarcinomas, hepatocellular carcinomas (*n* = 11), cholangiocarcinomas (*n* = 11), and high-grade differentiated, poorly differentiated and anaplastic thyroid carcinomas of follicular cell derivation (*n* = 10). Both DLL3 and SEZ6 were negative with only focal weak positivity for DLL3 in three tumors, a poorly differentiated colonic adenocarcinoma, a multifocal moderately to poorly differentiated hepatocellular carcinoma, and a moderately differentiated cholangiocarcinoma with weak to strong expression in 10 to 30% of the tumor cells (H-score ranging from 20 to 50; average 30). One steatohepatitic hepatocellular carcinoma was moderately positive for SEZ6 in 5% of the tumor cells (H-score 10). Among the 10 thyroid carcinomas, only two poorly differentiated oncocytic carcinomas were weakly positive in 10% of the cells for DLL3 (H-score 10), and only one of those tumors was moderately positive for SEZ6 in 5% of the cells (H-score 10).

## Discussion

This comprehensive analysis of DLL3 and SEZ6 expression across the spectrum of neuroendocrine neoplasia reveals distinct patterns that may have important implications for diagnosis, prognosis, and targeted therapy.

DLL3 expression was particularly prominent in high-grade neuroendocrine carcinomas, including lung NECs and Merkel cell carcinomas. This finding is consistent with previous studies reporting DLL3 expression in small-cell lung cancer and suggests a potential role for DLL3-targeted therapies in these aggressive tumors [4, 5, 11]. Medullary thyroid carcinomas showed strong expression of DLL3, as previously reported [4]. This observation may open new avenues for targeted therapy in this challenging tumor type. The identification of DLL3 in thymic NET is novel but not unexpected. Paragangliomas have also been reported to express DLL3 [4], and we confirm this finding. We are the first to report DLL3 expression in PitNETs and one parathyroid carcinoma. There was also positivity in one gastric and two appendiceal NETs. No expression was seen small bowel or colorectal NETs. As reported previously [4–6], some gastroenteropancreatic NECs were positive but so were a few pancreatic NETs, as reported previously [5]. These findings support the expanded application of bispecific antibody therapy for patients with NETs that are refractory to the current therapeutic approaches.

SEZ6 expression was more variable across different NEN types but was consistent and strong in medullary thyroid carcinoma as previously reported [10]. Notably, there was also strong staining for SEZ6 in two duodenal NETs that both expressed gastrin. We also identify staining in appendiceal NETs and frequent expression in pancreatic, ileal, appendiceal, and rectal NETs as well as PitNETs and thymic NETs. The expression of SEZ6 in paragangliomas is not unexpected. The biological significance of SEZ6 expression in these tumors warrants further investigation, but this common occurrence offers opportunities for novel approaches to the treatment of patient with refractory recurrent or metastatic disease.

The broad spectrum of expression levels in the various tumors raises questions about the level and pattern of expression that will predict potential response to therapy with targeted agents. We used the H-score to assess intensity and percentage of positive cells in our evaluation. There are minimal data as yet on which of these parameters will prove critical to clinical efficacy of these treatments.

The almost complete lack of staining in non-NENs makes these markers potentially useful in diagnosing neuroendocrine differentiation. However, further evaluation of the expression of these two immunomarkers in non-neuroendocrine neoplasms across other organs is warranted to assess their specificity and diagnostic utility. Importantly, their expression patterns could guide the selection of patients for clinical trials of novel therapies targeting these proteins.

In conclusion, this study provides a comprehensive overview of DLL3 and SEZ6 expression in the spectrum of neuroendocrine neoplasms, revealing distinct patterns across different tumor types and grades. The findings suggest potential applications for these markers in diagnosis, prognosis, and targeted therapy of NENs. Further research is needed to elucidate the functional roles of DLL3 and SEZ6 in neuroendocrine tumorigenesis and to evaluate the efficacy of targeted therapies in DLL3- and SEZ6-positive tumors.

## Data Availability

All data supporting the findings of this study are available within the paper.
